# Physical Activity and Sarcopenia in Community-Dwelling Older Adults with Long-Term Care Insurance

**DOI:** 10.3390/ejihpe11040114

**Published:** 2021-12-08

**Authors:** Masahiro Kitamura, Kazuhiro P. Izawa, Kodai Ishihara, Hiroaki Matsuda, Soichiro Okamura, Koji Fujioka

**Affiliations:** 1 Department of Physical Therapy, Fukuokawajiro Professional Training College, 2-1-13 Wajirooka, Higashi-ku, Fukuoka 811-0213, Japan; pt_masa0808@yahoo.co.jp; 2 Department of Public Health, Graduate School of Health Sciences, Kobe University, 7-10-2 Tomogaoka, Suma-ku, Kobe 654-0142, Japan; mhe1601@std.huhs.ac.jp; 3 Cardiovascular Stroke Renal Project (CRP), Kobe 654-0142, Japan; 4 Department of Rehabilitation, Sakakibara Heart Institute of Okayama, 5-1 Nakaicho 2-Chome, Kita-ku, Okayama 700-0804, Japan; 5 Department of Rehabilitation, Rifuru Yukuhashi Day Care Center, 379-1 Takase, Yukuhashi 824-0027, Japan; matmat1213@gmail.com (H.M.); okamura19830216@gmail.com (S.O.); kouji.0314yh@gmail.com (K.F.)

**Keywords:** accelerometer, care level, cut-off value, skeletal muscle mass index

## Abstract

The present study aimed to clarify the difference in physical activity (PA) due to sarcopenia in community-dwelling older adults with long-term care insurance (LTCI). This was a cross-sectional study that investigated data of 97 consecutive community-dwelling older Japanese adults with LTCI who underwent rehabilitation at one day care center in Japan from November 2018 to May 2019. Sarcopenia was determined according to criteria of the Asian Working Group for Sarcopenia. Unpaired *t*-test, Mann-Whitney U test, chi-square test and analysis of covariance were used to compare participant characteristics and clinical parameters between the older adults with and without sarcopenia. A receiver operating characteristic (ROC) curve was constructed to determine the cut-off value of PA for sarcopenia. The sarcopenia group (*n* = 20) had significantly lower body mass index (BMI), skeletal muscle mass index, gait speed, and PA than those in the no sarcopenia group (*n* = 28) (*p* < 0.05). After adjustment for BMI and sex, the sarcopenia group showed significantly lower PA than the no sarcopenia group. Findings showed that the cut-off value of PA indicating sarcopenia by ROC curve analysis was 1494.4 steps/day (*p* < 0.05); this value may aid in identifying sarcopenia in older adults with LTCI.

## 1. Introduction

The number of older adults is increasing worldwide [1], especially in Japan, where older adults comprise 28% of the population, the highest in the world, and this rate is expected to rise to 38% by 2065 [2]. In Japan, the long-term care insurance (LTCI) system was launched in 2000 with the aim of reducing the need for long-term care and the burden on family members who provide such care [3]. The Japanese LTCI system provides services according to certification at seven levels (support levels one to two and care levels one to five) according to the condition of the person’s disease and physical and cognitive function. The care level of LTCI requires more care than the support level of LTCI, and the higher the level, the more care is needed [3]. However, the number of older adults with LTCI is increasing year by year (reported to be 6.79 million in 2021) and the cost of LTCI is becoming a serious problem [3]. Older adults with LTCI have a higher mortality rate than those without LTCI [4], and the risk factors for the need for LTCI are known to be increased comorbidities and the presence of sarcopenia [5,6]. Sarcopenia is defined as a decrease in muscle strength and physical function in addition to a decrease in skeletal muscle mass with aging [7], and diagnostic criteria have been established by the Asian Working Group [8]. In Japan, the prevalence of sarcopenia in older adults is 7–11% [9,10] and is as high as 22–30% in older adults with low physical function [11,12].

Physical activity (PA) has also attracted much attention as a countermeasure for problems in older adults with LTCI. Lack of PA is a risk factor for frailty and dysfunction in this group [13]. Engaging in PA reduces mortality and the risk of disability [14] and can help to control the development of lifestyle-related diseases [15] in older adults. Increased PA in older adults has also been reported to be effective in improving sarcopenia [16]. In addition, systematic reviews report that PA has a protective role against the risk of sarcopenia [17,18].

However, there are few studies on the difference in PA due to sarcopenia in older adults with LTCI, who may be at increased risk of sarcopenia due to low PA, and this lack of previous related studies is problematic. Also, the diagnosis of sarcopenia is limited by the need for special equipment, a complex diagnostic method and medical examination opportunities by doctors [19]. Therefore, to prevent or improve sarcopenia in older adults, a simple assessment via daily monitoring and a cutoff value are needed to identify sarcopenia. We hypothesized that among older adults with LTCI, those with sarcopenia would have lower PA than those without sarcopenia. The purpose of this study was to clarify the difference in PA due to sarcopenia in older adults with LTCI and to calculate the cutoff value for PA to identify sarcopenia.

## 2. Materials and Methods

### 2.1. Design, Setting and Participants Flow

In this cross-sectional study, we investigated the data of 97 consecutive community-dwelling older Japanese adults with LTCI who underwent rehabilitation at one day care center in Japan from November 2018 to May 2019. We included participants over the age of 65 who were able to walk with or without aids and excluded participants in whom skeletal muscle mass index (SMI) and PA could not be measured and those with severe dementia. The investigators identified older adults with severe dementia, in whom measurement may have been problematic due to difficulties in communication.

**Figure 1:**
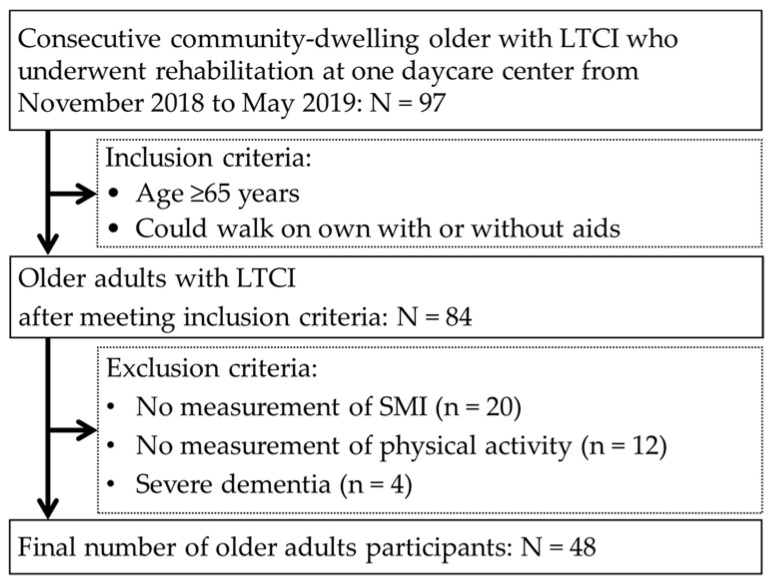
Participant flow.

Participant flow in the study is shown Figure 1. Of the 97 consecutive community-dwelling older adults with LTCI who underwent rehabilitation, 84 older adults who met the inclusion criteria were originally included in this study. However, 20 older adults in whom SMI could not be measured, 12 in whom PA could not be measured, and 4 with severe dementia were excluded. Ultimately, 48 older adults were the participants in this study.

The present study was approved by the Fukuokawajiro Professional Training College Ethics Committee (approval no. FW-20-01), and written informed consent was obtained from each participant.

### 2.2. Measures

Characteristics of the participants and clinical parameters investigated included age, sex, LTCI level, comorbidities, body mass index (BMI), SMI, and handgrip strength, one leg standing time, gait speed and steps taken per day as indices of PA. The measured data were investigated by two physical therapists from a review of the participants’ records.

Hand grip strength was assessed using a Smedley-type hand dynamometer (TKK5401, Takei Equipment Industry Co., Ltd., Niigata, Japan) [20]. The maximum effort was measured twice on each side, and the maximum value obtained was used.

To assess gait speed, the time required to walk 10 m at normal speed was measured with a stopwatch. The walking speed (m/s) was calculated from each participant’s 6-m walking time excluding 2 m at the beginning and at the end of the walk [21].

For one leg standing time, the standing time on one leg with eyes open was measured as the time this posture was held for up to 60 s. It was measured twice on the left and right sides, and the longest time was used [22].

To measure skeletal muscle mass, a multi-frequency electrical impedance meter (InBody 430, Biospace Japan, Tokyo, Japan) was use [23]. SMI was calculated by dividing the skeletal muscle mass of the left and right limbs by the square of the height [23].

The determination of sarcopenia was made according to the criteria of the Asian Working Group for Sarcopenia [8]. Sarcopenia is diagnosed when either or both handgrip strength of ≤28.0 kg in males and ≤18.0 kg in females and normal gait speed of ≤1.0 m/s are present, and SMI is ≤7 kg/m^2^ in males and ≤5.7 kg/m^2^ in females.

### 2.3. Long-Term Care Insurance

In the event of an older adult’s need for support or care, the LTCI level is determined by the LTCI committee in the city in which the person dwells. The support level of LTCI defines conditions that are expected to interfere with daily life, and the care level of LTCI defines conditions that require constant care in daily life, and services are available for each. Support level one is for people who are independent in activities of daily living but require some watching over for some of the instrumental activities of daily living such as shopping. Support level one is for people with reduced gait ability due to lower limb muscle weakness in addition to support level one. Care level one is for people who need care as part of their instrumental activities of daily living. Care level two is for people who need care as part of their activities of daily living. Care level three is for people who walk with aids or use wheelchairs for locomotion and need care for many of their activities of daily living. Care level four is for people who need a wheelchair for locomotion and cannot perform activities of daily living without care. Care level five is for people who are almost bedridden, have difficulty communicating, and cannot eat on their own [3]. These LTCI levels were investigated from the participants’ medical data by two physiotherapists.

### 2.4. Physical Activity

We measured the steps taken over a week using an accelerometer (Kenz Lifecorder EX, Suzuken Co., Ltd., Nagoya, Japan) to measure PA and computed the daily average [24]. All participants wore their accelerometer on a waist-level belt when waking up and removed it before bedtime. Steps are recorded by the accelerometer after pre-entry of age, sex, height, and weight data. After retrieval of each device, the recorded data were downloaded to a computer.

To calculate the sample size, the average daily steps in previous studies ranged from 5081 to 7009 steps (effect size = 0.73) [25]. We needed a minimum of 26 participants in each group with a significance level of 0.05, a power of 0.8, and an effect size of 0.7. In total, 65 participants were recruited for this study, taking into account a dropout rate of 20%.

### 2.5. Statistical Analysis

Participant characteristics and clinical parameter values are reported as percentages for categorical variables and as mean ± SD for continuous variables. The Shapiro-Wilk test was used to asses variable normality. The unpaired *t*-test for normally distributed continuous variables, the Mann-Whitney U test for continuous variables not normally distributed, and the chi-square test for categorical variables were used to compare participant characteristics and clinical parameters between the sarcopenia group and no sarcopenia group. Analysis of covariance was used to compare the differences in PA between the two groups. The covariates used were variables that showed a significant difference between the two groups, and sex, excluding factors related to sarcopenia criteria. A receiver operating characteristic (ROC) curve was used for the identification of sarcopenia and the area under the curve (AUC) was calculated. The Youden index was used to determine the cutoff value for PA indicating sarcopenia. For AUC values, >0.9 indicates high accuracy, 0.7–0.9 indicates moderate accuracy, and <0.7 indicates low accuracy [26].

A *p*-value of <0.05 was considered to indicate statistical significance. Statistical analyses were performed with IBM SPSS 25.0 J statistical software (IBM SPSS Japan, Inc., Tokyo, Japan).

## 3. Results

### 3.1. Characteristics of the Sarcopenia Group

The 48 participants were divided into the sarcopenia group and no sarcopenia group. Table 1 shows the characteristics of the sarcopenia group in the community-dwelling older adults with LTCI. Compared with those in the no sarcopenia group, these older adults had a significantly lower BMI, SMI, gait speed and PA than those in the no sarcopenia group.

### 3.2. Adjusted PA in the Sarcopenia Group

After adjusting for BMI, the sarcopenia group showed significantly lower PA (859.4 ± 371.3 vs 2526.1 ± 310.3 steps/day, F = 11.1, *p* = 0.002) than the no sarcopenia group. After adjusting for BMI and sex, the sarcopenia group still showed significantly lower PA (923.8 ± 375.8 vs 2480.1 ± 1849.4 steps/day, F = 9.3, *p* = 0.004) than the no sarcopenia group.

### 3.3. Cut-Off Value of PA for Sarcopenia

**Figure 2:**
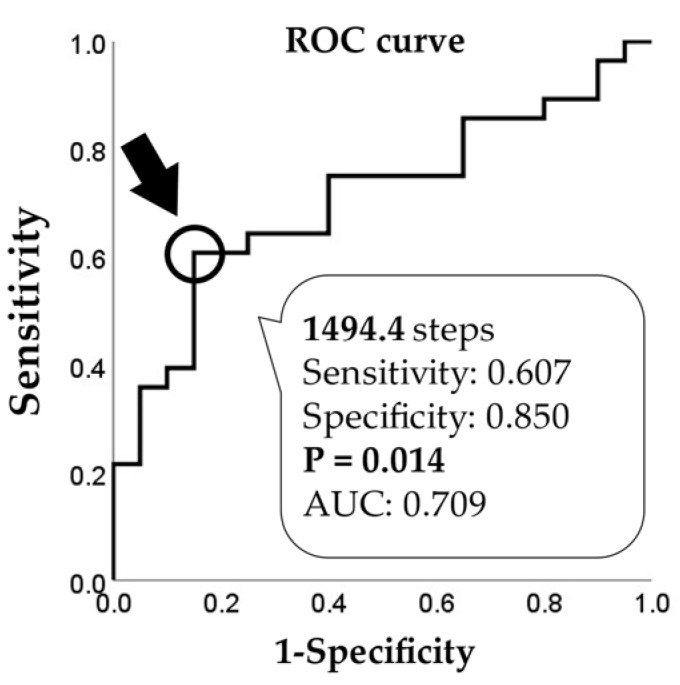
Cut-off value of PA for sarcopenia.

Figure 2 shows the cut-off value of PA indicative of sarcopenia as determined by the ROC curve.

## 4. Discussion

To our knowledge, this is the first report to show a difference in PA performed by older Japanese adults with LTCI in relation to the presence of sarcopenia. The results showed that the prevalence of sarcopenia in the older adults with LTCI was 41.7% and that the older adults in the sarcopenia group had lower PA than those in the no sarcopenia group. The cutoff value of PA indicating sarcopenia was 1494.4 steps/day.

### 4.1. Prevalence and PA of Sarcopenia

According to the Asian Working Group for Sarcopenia criteria 2019 [8], the prevalence of sarcopenia is reported to be 12–17% for older Japanese adults overall and 27% for older adults living in China [9,27]. However, the prevalence of sarcopenia among the older adults with LTCI in Japan is reported to be 48% [28]. Our results showed a prevalence of sarcopenia of 41.7%, which was similar to that in the previous study, and higher than that of the older adults without LTCI. After adjusting for BMI and sex, the average steps/day of PA in the sarcopenia group were significantly lower than those in the no sarcopenia group. Decreased PA is a known cause of sarcopenia [29,30], and previous studies have reported that skeletal muscle mass decreases in older adults due to decreased PA [31]. Further, a decrease in the number of steps can help to distinguish between frailty and non-frailty in older adults [32]. Therefore, it was suggested that older adults with LTCI with sarcopenia, which reflects the state of decreased muscle strength and skeletal muscle mass, had a lower PA than older adults without LTCI. However, the actual number of steps taken by participants in the sarcopenia group in this study may be underestimated due to their low gait speed [33]. Also, cognitive function and depression are known to reduce the number of steps, but this study could not investigate cognitive function and psychological indicators [34,35]. Considering these factors, it is necessary to carefully interpret the number of steps obtained in the sarcopenia group in this study.

### 4.2. Cut-Off Value of PA for Sarcopenia

The cut-off value to discriminate sarcopenia was 1494.4 steps/day, with the AUC value indicating moderate accuracy. The indicators of PA measured in this study are easily evaluated, can be easily understood by the participants, and are useful for predicting the risk of disease [25]. It has been reported that the number of steps needed for the prevention of depression is a minimum of 4000 steps/day and that to help prevent heart disease and stroke is a minimum of 5000–6000 steps/day [36,37,38]. Other studies reported that the number of steps/day taken by older adults with LTCI was 2000–3000 steps, similar to the number taken by participants in the present study [39,40]. The older adults with LTCI have certain cognitive and physical dysfunctions and take fewer steps than the older adults without LTCI [41,42]. Those who take less than 2000 steps/day are reported to be at high risk of non-independence and bedridden at home [25], and step counts in the older adults and those with chronic illnesses who have sarcopenia are lower than those in the older adults without sarcopenia [31,43]. Thus, the cutoff value of 1494 steps/day obtained in this study may be a reasonable index for discriminating sarcopenia in older adults with LTCI.

Furthermore, as one of the ways to improve sarcopenia, the evaluation and promotion of PA is recommended [13,14], and the effectiveness of programs using accelerometers has also been documented [44,45]. Therefore, for these programs to reduce the onset and exacerbation of sarcopenia, it may be important to monitor and clinically evaluate steps and increase PA in the fields of daily care and support.

### 4.3. Strengths and Limitations

This is the first study to determine the cut-off value for PA related to sarcopenia in older Japanese adults with LTCI. Following adjustment for BMI and sex, the PA of the older adults with LTCI and sarcopenia was significantly lower than that of the older adults with LTCI and no sarcopenia, with a cut-off value of 1494 steps/day indicating sarcopenia. This might be important information for the prevention and improvement of sarcopenia in older adults with LTCI.

There are some limitations in this study. This study was conducted in a single facility with a small sample size. Therefore, it was not possible to investigate the data segmented by sex. The causal relationship between sarcopenia and PA was not explained in this study due to its cross-sectional design. Factors relating to sarcopenia such as nutrition, psychological function and cognitive function were not examined. Further, the number of steps of participants with low gait speed may have been underestimated.

## 5. Conclusions

Among community-dwelling older adults with LTCI living in a day care center in Japan, those with sarcopenia showed significantly lower PA than those without sarcopenia. The cutoff value of PA to indicate sarcopenia was 1494.4 steps/day, which may be a useful value to aid in the identification of sarcopenia in older adults with LTCI. In addition, monitoring and clinical evaluation of steps may be required in the fields of routine care and support to mitigate the onset and exacerbation of sarcopenia. In the future, it will be important to accumulate data on the amount of PA of a greater number of participants and clarify the relationship between their amount of activity and sarcopenia. Although there are many limitations, this is a rare study investigating sarcopenia and PA in subjects who specifically require care and support. Monitoring and clinical evaluation of the number of steps may be useful in older adults who require routine care or support to mitigate the onset and exacerbation of sarcopenia.

## Figures and Tables

**Table 1:** Characteristics of the Sarcopenia Group.

	Sarcopenia			No Sarcopenia			*t*, Z or χ^2^ Value	*p* Value
	*n*= 20			*n* = 28				
Prevalence of sarcopenia, %	41.7			58.3				
Age, years	78.4	±	7.4	77.8	±	6.8	0.3	0.790
Sex, Male, %	40.0			25.0			1.2 ^a^	0.270
BMI, kg/m^2^	22.7	±	3.3	25.8	±	4.5	2.2 ^b^	0.025
SMI, kg/m^2^	5.6	±	0.8	6.5	±	1.0	3.2	0.002
LTCI level, %								
Support level 1	63.2			53.6			3.2 ^a^	0.530
Support level 2	10.5			25.0				
Care level 1	10.5			14.3				
Care level 2	10.5			7.1				
Care level 3	5.3			0.0				
Comorbidity, %								
Hypertension	65.0			78.6			1.1 ^a^	0.300
Diabetes	25.0			14.3			0.9 ^a^	0.350
Orthopedic disease	50.0			60.7			0.5 ^a^	0.460
Neurological disease	40.0			39.3			<0.1 ^a^	0.960
Heart disease	80.0			78.6			<0.1 ^a^	0.900
Cancer disease	20.0			14.3			0.3 ^a^	0.600
Physical function, Physical activity								
Handgrip strength, kg	19.2	±	7.2	21.8	±	8.6	1.1	0.268
One leg standing time, s	6.8	±	13.2	13.4	±	17.1	1.7 ^b^	0.088
Gait speed, m/s	0.70	±	0.18	0.93	±	0.30	3.1	0.004
Physical activity, step/day	1052.4	±	739.2	2388.3	±	2044.4	2.4 ^b^	0.014

Values are presented as mean ± standard deviation or %, ^a^ χ^2^ value, ^b^ Z value. BMI, body mass index; LTCI, long-term care insurance; SMI, skeletal muscle mass index.
